# Characterization of Oregano Essential Oil (*Origanum vulgare* L. subsp. *hirtum*) Particles Produced by the Novel Nano Spray Drying Technique

**DOI:** 10.3390/foods10122923

**Published:** 2021-11-25

**Authors:** Fotini Plati, Rigini Papi, Adamantini Paraskevopoulou

**Affiliations:** 1 Laboratory of Food Chemistry and Technology, School of Chemistry, Aristotle University of Thessaloniki, 54 124 Thessaloniki, Greece; fcplati@chem.auth.gr; 2 Laboratory of Biochemistry, School of Chemistry, Aristotle University of Thessaloniki, 54 124 Thessaloniki, Greece; rigini@chem.auth.gr

**Keywords:** oregano essential oil, encapsulation, nano spray drying, whey protein isolate, maltodextrin, morphology, antibacterial activity

## Abstract

Oregano essential oil (OEO), due to its wide variety of biological activities, could be a “green” alternative to chemical preservatives. On the other hand, the difficulties in its use or storage have turned researchers’ interest in encapsulation strategies as a way to face stability and handling issues. Fabrication of OEO-loaded particles, using nano spray drying technique (NSD) and whey protein isolate-maltodextrin mixtures (1:1, 1:3) as wall materials appears to be a novel and promising strategy. The obtained particles were characterized in terms of volatile composition, encapsulation efficiency, and physicochemical, molecular, morphological, and antibacterial properties. The results confirmed that encapsulation of OEO using NSD achieved high levels of powder recovery (>77%) and encapsulation efficiency (>98%) while assisting in the retention of the main bioactive compounds. The partial replacement of WPI by MD significantly affected particles’ physical properties. FTIR analyses revealed the possible structural stabilization of core and wall materials, while SEM verified the very fine size and spherical shape. Finally, antibacterial studies demonstrated their activity against *Escherichia coli* and *Staphylococcus aureus*, which is much stronger in comparison with that of pure OEO, proving the positive effect of NSD and particles’ potential in future food applications.

## 1. Introduction

During the last decades, people have switched to healthier habits and lifestyles, adopting green consumer behaviors and organic foods. Thereby, there is a great demand for natural, health-promoting preservation ingredients that can be used in the food industry in the place of chemical preservatives. Essential oils (EOs) obtained from aromatic plants could serve as such “green” alternatives since they have been generally recognized as safe (ESO, GRAS-182.20). Oregano essential oil (OEO) is widely renowned for its pleasant flavor, but it has also gained considerable attention because of its broad spectrum of biological activities, particularly the antioxidant [1,2,3,4] and antimicrobial [2,3,4,5] ones, mainly related to the presence of carvacrol and thymol [5].

However, the use or storage of EOs in their conventional form entails stability and handling concerns that can lead to oxidation and evaporation, finally affecting their properties [1]. Encapsulation is an alternative strategy, described by Jafari et al. (2008) [6] as a “unique way” to package materials in the form of micro- and nano-particles, that has been extensively used for bioactive compounds’ protection, preventing undesirable reactions and achieving controlled release and easier handling [1,4]. OEO encapsulation has been broadly studied with respect to the different techniques, such as spray drying [2,7,8,9,10,11,12,13], ionic gelation [1,14,15], nano-emulsification [16], nanoprecipitation [17], electrospraying [18], molecular inclusion [3,19], complex coacervation [20], etc., as well as the materials that can be applied. The generated OEO encapsulation forms, either liquid or powder, have demonstrated a wide range of encapsulation efficiency values (53.9–98.3%) and proved to be successful in strengthening OEO’s beneficial properties such as antimicrobial and antioxidant activity. Many of them have been also incorporated in real food systems (e.g., bread) [17] or in active packaging [21], as has been reported in other cases of EOs [22].

**Figure 1:**
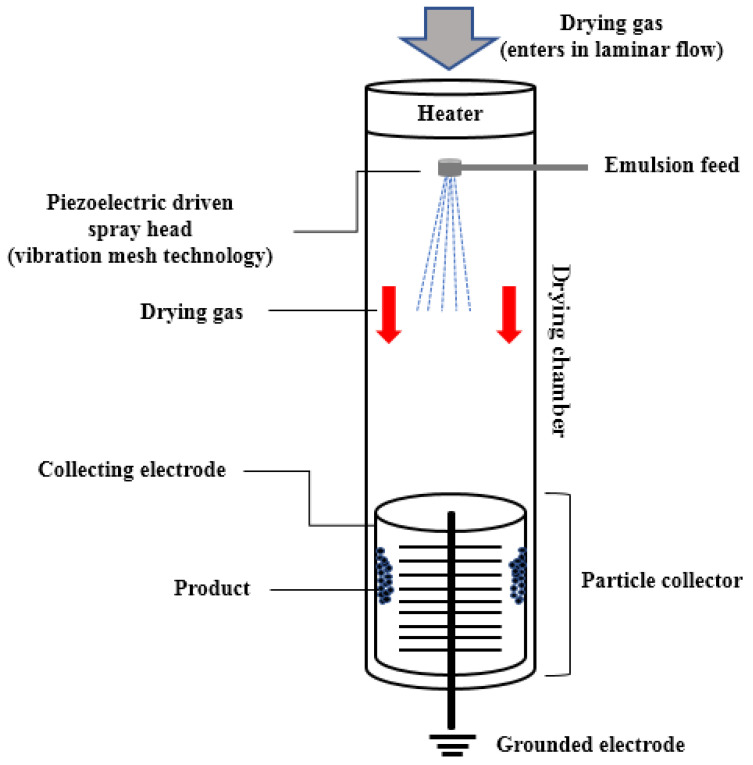
Schematic representation of Nano spray dryer B-90 and its functional principle function, as described by Lee et al. (2011) [23] and Arpagaus et al. (2012) [24].

Spray drying is the most investigated technology employed in OEO’s encapsulation, and is commonly used in the food industry due to its simplicity, rapidity, low cost, equipment availability, and efficient retention of volatiles [4,6]. Substantial efforts have been made to develop innovative technologies that further promote the protection of bioactive compounds. Among these, Nano spray dryer B-90 (NSD) is an emerging and promising technology that has been found in research fields from 2009. NSD’s technological novelty lies in the gentle laminar drying flow, the vibrating mesh spray technology, and the highly efficient electrostatic particle collector [23,24]. The vibration mesh technology is based on a piezoelectric actuator, which, through the production of ultrasonic waves, causes the vibration of a thin perforated stainless-steel membrane (i.e., spray meshes of 4, 5, or 7 μm) located in the head, leading to the generation of millions of droplets per second with a specific size and narrow distribution. The electrostatic particle collector consists of a stainless-steel cylinder (anode, electrode for collecting particles) and a grounded electrode of opposite charge, in the form of star inside the cylinder (cathode). High voltage is applied between the electrodes, the dehydrated particles are electrically charged, and, finally, they are deposited inside the cylinder. The drying principle is similar to that of conventional spray drying, but instrumental differences are found in the atomization system, drying flow principle (turbulent in MSD), and in the particle collector (common cyclone technology for particle collection during MSD depending on particles’ mass) (Figure 1). In fact, NSD’s potential is evident from the ability to modulate the particle’s size in submicron scale applying a single-step process, but also the low residence time that can preserve the active ingredients and even more the high levels of yield that are achieved (70–90%). Furthermore, the laminar drying flow achieves gentle heating, while the vertical configuration facilitates direct and straight-down collection of the particles in the collector [23,25]. Of great importance is, also, the fact that NSD technology gives the opportunity to apply inlet temperatures lower than 120 °C, in comparison with the higher ones that have been implemented in MSD applications (e.g., 150–190 °C) [2,7,9,10,11,12,13]. Due to the fact that it is intended for research purposes, drawbacks such as low throughput and increased processing hours have been reported [24,26]. Another issue that concerns NSD application is that viscous solutions can block the membrane’s (spray mesh) small holes, reduce the spray effectiveness, and, consequently, increase the processing time [25]. Finally, the high cost that accompanies this system may urge only high-end pharmaceuticals production [27].

An important step that determines the success of an encapsulation process is the choice of wall material. Good emulsification, film forming, and drying properties as well as high solubility and low viscosity are some of the characteristics that wall materials should meet [6,14]. Such properties are satisfied by whey proteins, which are among the most commonly used wall materials in a great variety of oils’ encapsulation [4], exhibiting excellent encapsulation properties, especially in combination with polysaccharides as it has been proved in the case of maltodextrin (MD) during spray drying encapsulation [28,29,30,31]. MD offers several advantages including high water solubility yielding solutions of low viscosity, while it is also able to enhance the spray drying process and reduce the tendency for agglomeration during storage [32].

The present work aimed at evaluating, for the first time, the potential of NSD B-90 to produce OEO’s particles. For this purpose, two combinations of whey protein isolate (WPI) and MD were chosen as encapsulating agents. In order to understand the role of NSD equipment in OEO’s encapsulation, morphological and physicochemical properties (encapsulation efficiency, oil retention, reconstitution properties etc.) of the spray dried products were examined. Moreover, the produced particles were examined in terms of their antibacterial activity against two pathogenic bacteria (disk diffusion method).

## 2. Materials and Methods

### 2.1. Materials

Oregano essential oil (*Origanum vulgare* L. subsp. *hirtum*) was kindly donated by ATHINA^®^ (Kiedrich, Germany). Whey protein isolate (WPI) (natural, unflavored, lecithin content <3%, 91% protein) from bovine milk was a product of Nestle (Frankfurt, Germany). Food grade maltodextrin (DE 17-19.9) was from Syral (Aalst, Belgium). Two bacterial strains, *Escherichia coli* (XL1 (Stratagene)) and *Staphylococcus aureus* (NCIM 2079), were used in the study for antibacterial activity testing and were stored at −80 °C. All other chemicals used were of analytical grade. Deionized water was used for the preparation of all solutions.

### 2.2. Preparation of Feed Emulsions

Initially, solutions of coating matrices were prepared by dispersing the powders in deionized water. WPI solution was prepared by mechanical stirring (IKA, Kuala Lumpur, Malaysia) for more than five hours followed by magnetic stirring overnight to ensure full hydration. MD was dissolved in water under magnetic stirring and kept overnight at 4 °C. Oil-in-water emulsions were prepared by gradually adding OEO (5% *w*/*w*) into WPI solution under continuous mechanical stirring at 600 rpm. The produced crude emulsion was further homogenized by using Ultra Turrax homogenizer (T25 Basic IKA LabortechNK, Malaysia) operating at 13,500 rpm for 5 min, followed by ultrasonication (130 W/cm^2^) for 2 min with the aid of a UP100H ultrasonic apparatus (Hielscher, Germany). An appropriate amount of MD solution was then added to the emulsion so that to give rise to two different WPI:MD ratios (1:1, 1:3) of 20% (*w*/*w*) final wall material concentration (Table 1). The final emulsion was then exposed to ultrasonication (130 W/cm^2^, 2 min) to enhance homogenization.

#### Emulsion Characterization

Emulsions were characterized in terms of their stability against coalescence and creaming following storage at 25 °C for four days, i.e., the time needed for a batch to be spray dried (100 cm^3^). Immediately after preparation, 10 cm^3^ of each emulsion was poured into a glass vial with screw cap and phase separation was visually assessed. A laser diffraction-based Malvern Mastersizer 2000 (Malvern Instruments, Malvern, UK) analyzer was used for the determination of the emulsions’ droplet size distribution (refraction indices: 1.50 and 1.33 for emulsion and water, respectively). Polydispersity index (*PDI*) was calculated according to Equation (1):

(1) $$PDI=\frac{d_{90}-d_{10}}{d_{50}}$$

where, *d*_10_, *d*_50_, *d*_90_ are the particle diameters for 10%, 50%, and 90% of the cumulative volume, respectively.

### 2.3. Production of Microparticles

The formed emulsions were subjected to drying using a Nano spray dryer B-90 (BÜCHI Labortechnik AG, Flawil, Switzerland) with the long version of the dry chamber, using a nozzle with a mesh pore diameter of 5.5 μm. Based on preliminary experiments to produce ultrafine powder, the samples were diluted 3 times with deionized water before spray drying, as has been already reported by Hu et al. (2016) [33] and Venerada et al. (2018) [34]. The inlet temperature was 100 °C, the gauge pressure was set at 23 mbar, relative spray rate at 60%, peristaltic pump speed at mode 1, and the air flow at 7.8 m^3^/h, while the outlet temperature ranged between 24 and 40 °C. The final product was collected from the surface of the cylindrical electrode using a rubber spatula, transferred to a plastic vial with a screw cap, and kept in a desiccator containing silica gel at room temperature (4 °C for antibacterial activity testing) until it was studied. For each treatment, powder recovery (%) was estimated from the ratio of the mass of the powder obtained after spray drying and the solid content of the initial feed emulsion.

### 2.4. Analysis of Encapsulated Oregano Essential Oil

#### 2.4.1. Total and Surface Oil Determination

The determination of total oil (TO) content was carried out in triplicate by Clevenger hydrodistillation method [7]. Approximately 5 g (±0.001 g) of the sample was dissolved in 250 cm^3^ deionized water in a 500-cm^3^ round-bottomed flask. The flask was stoppered, manually shaken for ~2 min in order to break down the clumps and facilitate dissolution, and a few boiling stones and ~0.5 cm^3^ of a silicon antifoam oil were added. The Clevenger apparatus was then connected to the top of the flask and, after the liquid inside the flask came to boil, allowed to distil for 3 h. The volume of the oil, read directly from the graduated oil collection arm, was converted to oil mass by multiplying with the oil density (0.948 g/cm^3^ at 20 °C), determined gravimetrically at 20 °C. Surface oil content (SO) was determined based on Hernández-Nava et al. [20]’s procedure with minor modifications. Briefly, 0.5 ± 0.001 g of powder was added into 20 cm^3^ hexane and the mixture was stirred for 15 s (80 rpm). The oil-containing hexane was filtered through a filter paper (pore size 2–3 μm), followed by centrifugation at 2500× *g* at 25 °C for 10 min. Hexane was removed from the extracted oil by using a rotary evaporator and the mass of the extracted oil was measured gravimetrically after further solvent evaporation under a steady stream of nitrogen. The extracts of the total and surface oil were stored at 4 °C until gas chromatographic analysis.

#### 2.4.2. Determination of Oil Retention and Encapsulation Efficiency

Oil retention (*OR*) was defined as the ratio of total oil in the final powder to that of the initial oil load and was calculated as follows (Equation (2)) [10]:

(2) $$OR\;\left(\%\right)=\frac{\mathrm{TO}}{\mathrm{initial}\;\mathrm{oil}\;\mathrm{load}\;}\times 100$$

Encapsulation efficiency (*EE*) was assessed by taking into account the total and surface oil content of the powders based on the Equation (3) [10].

(3) $$EE\;\left(\%\right)=\frac{\mathrm{TO}-\mathrm{SO}}{\mathrm{TO}}\times 100$$

#### 2.4.3. Gas Chromatographic Analysis of the Major Constituents of the Encapsulated OEO

The identification of volatile compounds in pure OEO (solution 2% *w*/*v* in dichloromethane) was performed on an Agilent 6890A GC-MS coupled with an MSD 5973 mass spectrometer (Palo alto, CA, USA), operated in electron-impact ionization (EI) mode with a mass scan range from 35 to 350 *m*/*z* (2 scans/s), at 70 eV. Chromatographic separation was achieved using a DB-WAX capillary column (60 m × 0.32 mm i.d. × 0.25 μm film thickness, Agilent Technologies, Wilmington, DE, USA) with helium as a carrier gas (2 cm^3^/min). Injection volume was 2 μL using a split ratio of 1:25. The oven temperature was initially set at 40 °C (5 min), increased to 100 °C by 15 °C/min, then to 140 °C by 5 °C/min, and finally to 230 by 15 °C/min, whereupon it was maintained (5 min). Injector and detector temperature was kept at 240 °C. The EO components were identified by comparison of their Kovats retention indices (KI) (relative to a series of C7-C30 n-alkanes, Sigma-Aldrich, Laramie, WY, USA) and mass spectra with those of standard compounds or those reported in the literature [5,35] or provided by the NIST (version 2.0g, 2011) mass spectral library.

Evaluation of the main volatile compounds of pure OEO and the samples recovered after TO and SO determination (as described in Section 2.4.1.) was accomplished with an Agilent 6890A (Palo alto, CA, USA) gas chromatograph equipped with a split-splitless injector and a flame ionization detector (FID). The samples were analyzed on a TR-FAME capillary column (60 m × 0.25 mm i.d. × 0.25 μm film thickness, ThermoFisher Scientific, Bellefonte, PA, USA). Chromatographic conditions were the same as in GC-MS analysis. Diluted samples (2% *w*/*v* in dichloromethane) were injected manually, and the GC split ratio was 1:25. The identity of the major compounds was confirmed by coinjection of standard compounds, while the relative amount of each of them was expressed as the percentage of the total amount of volatiles in the analyzed sample. The samples were evaluated in triplicate (CV < 5%).

### 2.5. Physicochemical Characterization of the Spray Dried Particles

#### 2.5.1. Moisture Content

The moisture content of the dehydrated samples was determined gravimetric at 103 ± 2 °C according to AOAC [36].

#### 2.5.2. Bulk Density

Bulk (*ρ_b_*) density was determined according to Plati et al. (2019) [37]. Powder samples were loosely weighed into a 10 cm^3^ graduate cylinder until the 10 cm^3^ mark and the density was calculated by dividing the sample weight by the volume.

#### 2.5.3. Reconstitution Properties

Wettability and dispersibility of the powders were determined using the method described by Plati et al. [37]. For wettability, 0.1 ± 0.001 g of powder were spread over the surface of distilled water (100 cm^3^) and the time taken for all powder particles to be completely wetted on the water’s surface was recorded. For dispersibility, 0.5 ± 0.001 g of powder were weighed, and the determination was accomplished after water addition (5 cm^3^), agitation with a spoon (15 s), sieving (pore diameter: 650 μm), and drying (103 ± 2 °C) until constant weight.

#### 2.5.4. Hygroscopicity

Hygroscopicity was determined according to the method proposed by Cai and Corke [38] with some modifications. Approximately 0.5 g of each sample were placed in a glass desiccator containing a saturated solution of NaCl (75% RH) for 1 week at room temperature. Hygroscopicity was expressed as g of moisture per 100 g dry solid (g/100 g).

### 2.6. Scanning Electron Microscopy

A JSM-7610F Plus JEOL (Tokyo, Japan) Scanning Electron Microscopy (SEM) equipped with an AZTEC ENERGY ADVANCED X-act EDS (Oxford, UK) analyzer, operating at 15.0 kV, was used for the examination of the external structure of the produced microcapsules. The powders were attached on the specimen stubs and coated by carbon using a JEE-4X vacuum evaporator to achieve electrical conductivity. Micrographs were taken at a magnification of ×1000.

### 2.7. Fourier-Transform Infrared Spectroscopy Analysis

The FT-IR measurements were assessed by Shimadzu FTIR spectrometer (IRAffinity-1 spectrometer, Shimadzu Corporation, Kyoto, Japan). The different samples (2 mg) (WPI, MD, pure OEO, WM, W3M and the corresponding empty spray dried particles WM_blank, W3M_blank) were mixed with KBr (180 mg) to form discs and FTIR spectra (64 scans at a resolution of 4 cm^−1^) were collected over the range of 4000–400 cm^−1^ in the absorbance mode and baseline was corrected with the aid of the software.

### 2.8. Determination of the Antibacterial Activity of the Spray Dried Particles

The antibacterial efficacy of the spray-dried powders against two microbial species (*E. coli*, *St. aureus*) was evaluated according to the agar disc diffusion method [39,40]. KBr discs of 13 mm diameter were integrated with 25, 50, 75, 100, and 125 mg of the samples. The OEO concentration range, determined on the basis of the TO values obtained by applying the Clevenger hydrodistillation method, was from 2.8 to 13.7 and from 2.5 to 12.4 mg for the WM and W3M samples, respectively. Then, 0.1 milliliter of 1.5 × 10^8^ CFU/cm^3^ of each bacterial suspension was spread evenly over the agar (LB agar) petri dishes’ surfaces and afterwards discs were dispensed. The antibacterial activity was evaluated by measuring the diameters of the inhibition zones (mm) after 24 h incubation at 37 °C. These treatments were compared with discs loaded with empty spray dried particles, produced following the same steps as OEO-containing particles, and used as antibacterial activity controls to guarantee that wall materials had no antimicrobial effect.

### 2.9. Statistical Analysis

Results were expressed as means and standard deviation of three or five assays carried out in each method. SPSS 25 (SPSS Inc., Chicago, IL, USA) software was used to evaluate significance of differences through unpaired samples *t*-test. Significance level was set at *p* < 0.05.

## 3. Results and Discussion

### 3.1. Characterization of Feed Emulsions

**Figure 2:**
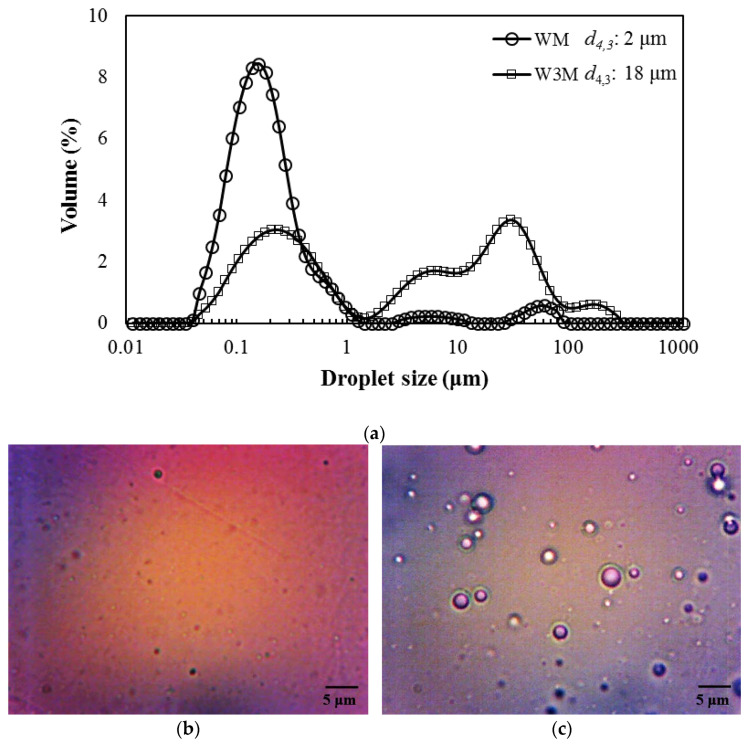
Droplet size distribution (**a**) and photomicrographs (**b**): WM, (**c**): W3M) of OEO emulsions.

Emulsion characteristics, such as droplet size distribution and stability, have a significant effect on encapsulation efficiency and consequently in products’ quality parameters (morphology, oxidation stability) [13,14,15]. Emulsions of smaller droplet sizes, apart from their characteristic stability, are strongly correlated to particles of higher *EE* due to the more efficient entrapment of the active substances within the wall materials, and their prevention from breaking down during atomization. The higher the *EE*, the lower the content of surface oil and thus better product stability due to less oil exposure to atmospheric oxygen and limited particle agglomeration [6]. OEO’s emulsions, prepared at a given EO concentration and two different WPI:MD ratios (1:1 and 1:3), exhibited multimodal droplet size distribution. Evidently, the droplet size distribution was affected by wall material ratio variation as replacement of WPI with MD caused a rise in droplet size (in terms of *d*_4,3_) from 2 to 18 μm (Figure 2). A similar trend has been also noted by Bae and Lee (2008) [16] and Shamaei et al. (2017) [17], during emulsification of avocado oil using WPI and MD and walnut oil using skim milk powder (SMP) and MD, respectively. This behavior was ascribed to the excellent emulsifying properties of WPI that are reduced upon maltodextrin incorporation increase in wall materials, a polysaccharide characterized by poor surface activity and low emulsifying capacity. Besides, the emulsion with the low WPI content (W3M) exhibited a higher PDI value than the WM (i.e., 11.4 to 2.9), thus confirming the higher degree of size distribution and polydispersity. Similar size and PDI values with those of WM system was also observed in the case of a ginger EO emulsion using the same proportion of WPI and MD [18]. The stability measurement indicated that both emulsions were kinetically stable since there was no visible phase separation after 4 days storage at room temperature (Figure S1). Stable emulsions have been related to considerable ability of flavor retention [19].

### 3.2. Powder Recovery, Oil Retention and Encapsulation Efficiency

Powder recovery represents the dry material recovered after encapsulation process, very useful information in the case of EOs as they are characterized by high volatility especially during drying. WM particles exhibited significantly higher powder recovery value (81.6%) in comparison with that of W3M (77.9%) (*p* < 0.05), indicating that a higher percentage of OEO was entrapped. Similar percentages (>70%) have been also reported by other researchers who have applied the NSD technique to produce protein nanoparticles [23,24]. At the same time, lower enough powder recovery levels have been reported for OEO encapsulation implementing the conventional spray drying technique (MSD) using either hydroxypropyl methyl cellulose, MD and colloidal silicon dioxide (31.1–52.6%) or MS (modified starch), GA (gum Arabic), and MD (~60%) or MD-GA mixture (20.6–89%) [2,8,9].

The most crucial parameters concerning the encapsulation process by means of spray drying are the retention of volatiles, the improvement of *EE*, and the prolongation of the core’s shelf life [29]. All of them are related to the amount of oil loaded into the particles (total oil content) as well as to the amount of oil that is not entrapped (surface oil content). At first sight, the total and surface oil contents were significantly influenced by the different protein to polysaccharide ratio of the encapsulating matrices (*p* < 0.05). As found, the replacement of protein with polysaccharide brought about a reduction in the quantity of EO entrapped into particles, increasing at the same time the quantity of EO that remained on the particles’ surface (Table 2). The latter is closely associated with the initial emulsions’ droplet size, justifying the effect of the increased droplet size of W3M sample onto the surface oil content, and it is considered of utmost importance for the storage stability of encapsulated cores [6]. What, however, is worth emphasizing in this place is that, although significantly different, the surface oil content values ranged in very low levels for both samples (0.2 and 0.9% for WM and W3M, respectively) in comparison with the values reported, e.g., in the cases of lime EO, avocado, and fish oil encapsulation using whey proteins in combination with MD by applying MSD (2.2–3.3, 11.4–15.8, and 6.5%, respectively) [28,30,31].

As it can be seen in Table 2, OEO’s retention (*OR*) was 54.9% and 49.4% for WM and W3M, respectively. *OR* values were within the range reported in relevant studies, i.e., from 33.1% to 77.4% in OEO encapsulation in various combinations of gum Arabic, modified starch, and MD with MSD [10]. Concerning OEO’s encapsulation efficiency (*EE*), this was found to be as high as 98.1% and 90.6% for WM and W3M, respectively. Similar findings (98.3–99.7%) have been reported by Partheniadis et al. (2019) [1] using a combination of GA, MD, and MS, while Toledo Hijo et al. (2015) [11], Baranauskiene et al. (2006), [13] and Da Costa et al. (2013) [10] noticed lower levels using MS-GA (86.2%), WPC, and SMP (71.8% and 80.2%) or GA, MS, and MD (85.3–93%) mixtures, respectively, by applying conventional spray drying. Lower levels have been also observed in the case of applying the nano-spray-drying technique for the encapsulation of corn oil in either MD-egg yolk-GA or MD-egg yolk-carboxymethyl cellulose mixtures (*EE* 46.1% and 80%, respectively) [37]. Additionally, nano spray drying proved to be sufficient to cover a great amount of core material at both biopolymer ratios tested, ensuring that encapsulation process by this technique was highly efficient in the case of OEO. Furthermore, these values were remarkably higher even when compared with different methods found in the literature for OEO encapsulation, such as nanoemulsification (82.8%) [16], ionic gelation (13.6–31.4%) [41], nanoprecipitation (79.6%) [17], and electrospraying (70.1–79.6%) [18]. Based on the data reported, we can say that nano spray drying, although time-consuming, excels relative to the aforementioned techniques in terms of *EE*. However, technical problems, such as blockage of membrane’s holes, frequently appeared, thus implying their rapid deterioration and increasing costs that should be of concern in future applications.

### 3.3. Composition of Oregano Essential Oil before and after Encapsulation

Identification of volatile constituents of original OEO was accomplished by GC-MS analysis, and evaluation of their selected volatile compounds was performed by GC-FID analysis. Thirty six compounds were identified in accordance with the previously reported chemical composition of OEO (*O. vulgare* L. subsp. *hirtum*) [5,35]. Carvacrol, an important compound which is responsible for most of OEO’s properties, was identified in the literature as the main component [5,35].

The compositional changes that may occur following encapsulation, especially when it takes place under high temperatures, are considered to be of significance. Table 3 shows the percentages of major compounds present in OEO extracted from the produced particles in comparison with those before the encapsulation process. Carvacrol was still the major OEO constituent, detected at 86.6% and 86.1% for WM and W3M, respectively, significantly higher levels compared with that of pure OEO (80.03%). A similar tendency was also ascertained for β-caryophyllene content. This could be explained by the losses of the most volatile compounds (boiling points between 167–183 °C (myrcene, a-terpinene, γ-terpinene, p-cymene) <238 °C boiling point of carvacrol), as has been also noted for the increase of carvacrol content in the case of OEO encapsulation in β-cyclodextrin or in GA-MD-MS system using MSD [3,7]. Likewise, a decrease in the content of γ-terpinene, p-cymene, terpinen-4-ol, and thymol was apparent (*p* < 0.05), while other compounds present in the original EO, such as myrcene and α-terpinene, were not identified after encapsulation, albeit temperature conditions were remarkably milder compared to those applied in the case of MSD. The insufficient entrapment could be ascribed to the volatility and sensitivity of these compounds or/and to the exposure to adverse factors, such as oxygen and light, that may exist during the particles’ fabrication. The same trend has also been observed in other studies of encapsulated OEO [3,7,10,13]. Regarding surface oil content, traces of the least volatile compounds (such as thymol and carvacrol) were found.

### 3.4. Physicochemical Characterization of the Spray Dried Particles

The determination of particles’ moisture content is of great importance as it is strongly related to their quality and shelf life [10]. In this work, moisture values of 9.2 and 8.2% (*p* > 0.05) were obtained for WM and WM3 powder samples, respectively, which are considered relatively high in comparison with other OEO encapsulates acquired via the conventional spray drying method using various wall materials, i.e., from ~3% to 5% after encapsulation in gelatin, GA, Tween 80, β-cyclodextrin [12], in GA, MD, MS [7], and in different mixtures of MD-GA [9]. Similarly high levels of moisture content have been also pointed by Stavra et al. (2021) [26] during lemon juice drying. Bearing in mind that operational conditions are mainly responsible for the products’ moisture content [30], the mild inlet temperature that NSD applies could possibly explain the appreciable amount of moisture observed in our case. The moisture content enhancement at low inlet temperatures has been also evidenced by Baranauskaite et al. (2016) [9], Botrel et al. (2012) [8], and Shamaei et al. (2017) [42], who attributed this behavior to the slower and less efficient water evaporation that takes place at these conditions. Another potential explanation could be the dextrose equivalent of MD, since it has been demonstrated that maltodextrins with higher DE result in products with higher moisture content [43].

**Figure 3:**
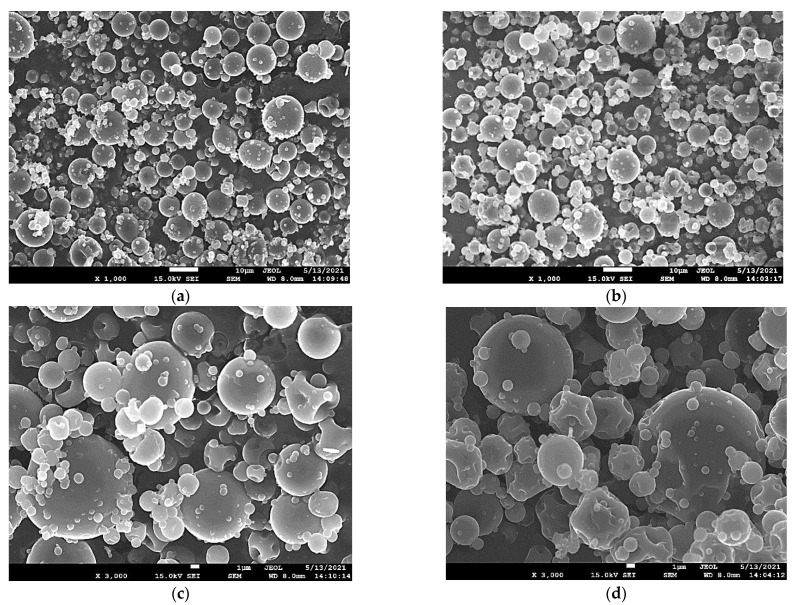
SEM micrographs of OEO nanoparticles using different wall material ratios (WPI:MD) 1:1 (**a**,**c**) and 1:3 (**b**,**d**).

Bulk density depends on chemical composition, moisture content, particle size, and morphology of the powder, and it is strongly related to processing conditions and procedures. It is about a property with major impact on packaging, reconstitution, and retailing characteristics [42]. The increase of MD content in the wall material had a significant effect on bulk density (WM: 0.17 g/cm^3^, W3M 0.20 g/cm^3^) (*p* < 0.05). Similar findings were also reached for avocado oil’s encapsulation via MSD by Bae and Lee (2008) [28] and were associated with a more agglomerated particle appearance, as it also seemed in our case (Figure 3b,d), resulting in a more compact arrangement of powder particles. Higher values, however, have been reported by applying the conventional spray drying approach, by Botrel et al. (2012) [8] for OEO (0.34–0.45 g/cm^3^) as well as by Kausadikar et al. (2015) [44] for lemon EO (0.38–0.47 g/cm^3^) encapsulation using GA, MD, and MS mixture. The lower levels obtained in this research mean that more air is occluded within powders, possibly due to the wide range of particles’ size as SEM micrographs revealed (Figure 3).

Reconstitution properties, such as wettability and dispersibility, uncover the particles’ behavior upon coming to contact with water. The ability of the powder particles to overcome the surface tension that develops among the interfaces involved is characterized as wettability [9]. It is obvious from Table 2 that OEO particles with lower WPI content (W3M) showed better wettability (5.2 min) in water than that of higher (7.3 min). The improvement of wetting time, as a result of the increase of MD content, is closely related to the existence of more hydrophilic groups that facilitate the accessibility and penetration of water into the powder particles [28,30]. The reverse effect of WPI contribution is in line with the presence of large hydrophobic chains in its structure [29]. The measured values are within the range (3.88–12.22 min) corroborated by Baranauskaite et al. (2016) [9], who examined the impact of inlet temperature and GA-MD ratio on OEO’s encapsulation via MSD, while they are superior to those of OEO encapsulates produced by different combinations of GA, MD, and MS using MSD (11–22 min) [10]. Factors that affect the capacity of wettability of a powdered material are, except of chemical and structural properties of wall materials, the capillary effects and the morphological characteristics [10]. So, particle agglomeration, shown in Figure 3, could be responsible for the satisfying wetting characteristics of the studied powders as the liquid penetrates more easily into the pores of particles’ arrangement [28]. Moreover, both samples exhibited moderate ability to disperse in water upon gentle stirring with the W3M (64.2%) being more capable than the WM one (57.1%) (*p* < 0.05). Similar values (55.6–64.8%) have also been reported for lemon juice samples dehydrated by using NSD and were attributed to the smaller particle size and, consequently, the larger specific surface area [26]. In accordance with the wettability results, the differences between the samples could be ascribed to the more hydrophilic nature of MD molecules. Lastly, resembling hygroscopicity values (~15%) were observed for both samples, lower enough compared with other investigations concerning either OEO (22.3–26.3%) or carvacrol (33.4%)-containing particles produced by the MSD method and stored under high RH conditions [23,39]. These values could be attributed to their high moisture content (~8–9%), which results in a lower water concentration gradient between the sample and the surrounding environment [45].

### 3.5. Scanning Electron Microscopy (SEM)

Figure 3 presents the external morphological features of powders produced with different WPI-MD ratios via NSD. The particles were well-formed with spherical shapes, smoothed surfaces, without visible pores or cracks, and their diameter range was between ~0.4 and 10 μm. Dents were barely observed on the surface, strongly associated with the wall-forming materials’ nature and the applied encapsulation method. The shrinkage that takes place during drying and cooling processes could be responsible for the formation of such surface characteristics [11]. These imperfections are more visible in the micrograph of W3M particles (Figure 3b, red arrows), somewhat interpreting their slightly lower *EE* in comparison with the WM one (Table 2). Dents are common in spray-dried particles, while similar structures have been also observed in many innovative NSD applications [23,26,37] as well as in the case of essential oil encapsulation in various matrices with the use of MSD [12,13,31].

Furthermore, the micrographs clearly revealed the existence of agglomeration among the individual spray dried particles, which could be partially attributed either to the presence of OEO traces onto their surfaces or to the increased moisture content. This behavior is comparable to that of other encapsulated oils (e.g., by NSD or MSD) as commonly reported in the literature [7,28,37]. Interestingly, by increasing the MD content the particles exhibited a more compact and tightly bound structure as that noted in the case of avocado oil encapsulation, justifying thus the corresponding changes in bulk density [28]. What is worth emphasizing in this place is that in both tested samples there were no collapsed or damaged particles, which ensures the presence of a resistant structure that will not compromise the stability of the encapsulated OEO.

### 3.6. FTIR Analysis

**Figure 4:**
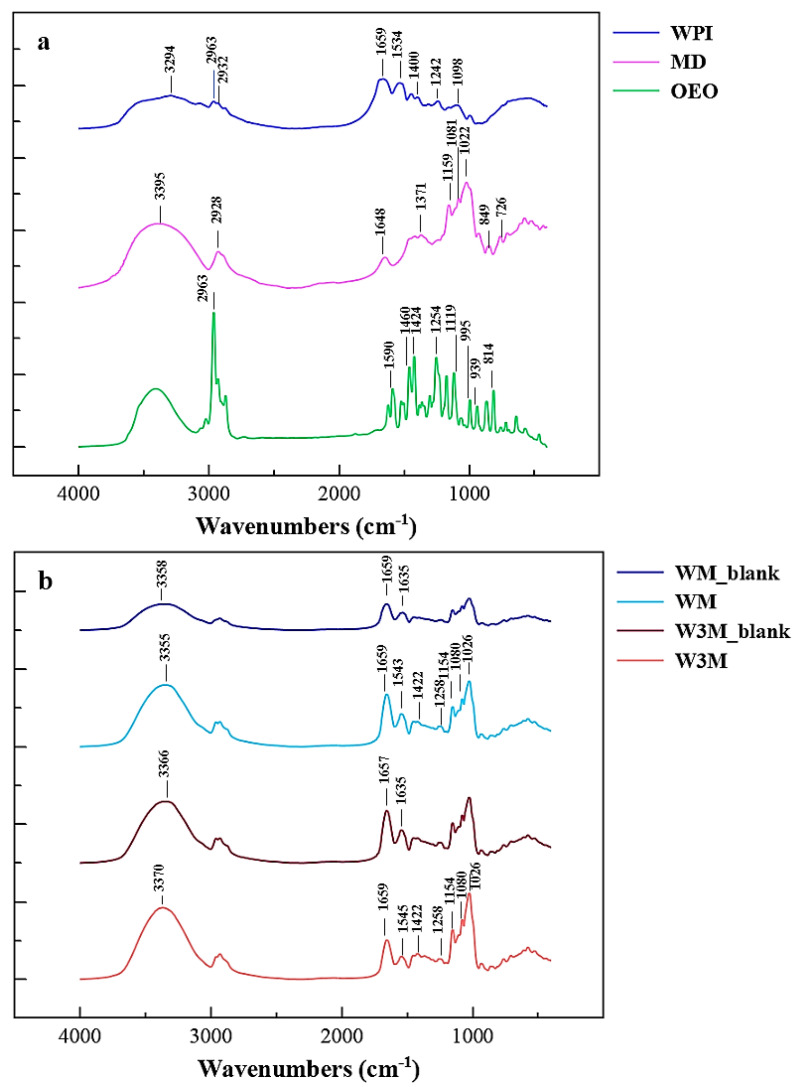
FTIR spectra of (**a**) raw wall materials (WPI, MD) and pure OEO and (**b**) particles without (WM_blank, W3M_blank) and with OEO (WM, W3M) produced using different wall material ratios.

FTIR analysis was performed to investigate the nature of interactions between the carrier materials and OEO during emulsification and following NSD. Figure 4a shows the spectra of all the components that take part in the encapsulation process. The spectrum of WPI revealed the presence of amide A at 3294 cm^−1^ due to the stretching vibration of N–H bond. The intense broadening of this peak is due to the hydroxyl group stretching vibration that, in combination with amide A, participates in the development of hydrogen bond. C-H stretching was observed at 2963 and 2932 cm^−1^, primary and secondary amino groups ascribed at 1659 and 1534 cm^−1^, respectively, while the band at 1400 cm^−1^ represents N–H bending and C–N stretching vibrations [46]. The peak at 1098 cm^−1^ is related to C–O–C stretching vibrations, while at 1242 cm^−1^ the peak is mainly the result of borderline between the random coil and β-sheet secondary structure present in WPI [47]. MD typical bands are assigned to O–H bonds at 3395 cm^−1^, C–H stretching at 2928 cm^−1^, C = O stretching at 1648 cm^−1^, O–H bending 1371 cm^−1^, C–O stretching and C–O–H bending at 1159, 1081, and 1022 cm^−1^ and skeletal vibrations of the pyranoid ring at 929, 849, 762, 710, and 577 cm^−1^ [48]. With respect to OEO chemical composition, the spectrum revealed peaks at 2963 cm^−1^ that indicated the presence of the aromatic C–H stretching vibration, at 1590 cm^−1^ assigned to N–H bending, at 1460 cm^−1^ where the CH_2_ bending is detected, and at 1424 cm^−1^ due to the C–C stretch of the aromatic ring of carvacrol, the major OEO component [15,19]. The peak at 1254 cm^−1^ is strongly related to the characteristic absorption of the C–O–C stretching vibration, while the characteristic peak at 939 cm^−1^ suggests the absorption of the C–H ring [19]. Other peaks that have been associated with the presence of carvacrol, confirming the GC-MS results, are 814, 995, 1119, and 1175 cm^−1^ [20].

Concerning the spectra of OEO-loaded particles, the presence of characteristic peaks of both wall materials is obvious (Figure 4b). The contribution of maltodextrin significantly altered the intensity of absorption at 1080, 1154, and mainly at 1026 cm^−1^ due to C–O–C groups, while the effect of WPI presence was obvious at 1659 (amide I, C = O stretching vibration) and 1543 or 1545 cm^−1^ (amide II, NH bending). Besides, the aforementioned OEO peaks could not be clearly seen in the spectra of OEO-loaded particles at the same wavenumbers, as they were almost completely obscured by the very intense and broad WPI-MD bands. These results showed that no substantial modifications or interactions took place between OEO and wall materials or that the OEO mass per mass of particles was too small to be detected. However, slight shifts or disappearances of some peaks at specific wavenumbers were distinct upon comparison of the spectra of OEO-loaded particles with those of the same composition but without containing OEO (i.e., WM_blank, W3M_blank) or that of WPI, as in the case of amide II shifts (1543 and 1545 cm^−1^ for WM and W3M, respectively) and amide III disappearance, respectively. At the same time, the existence of characteristic absorption peaks at 1422 and 1258 cm^−1^ in the spectra of both OEO-containing encapsulates, in combination with their absence in the corresponding blank samples, proves the presence of OEO. Additionally, the augmented intensity of the region between 3000 and 3600 cm^−1^ in powder samples with and without OEO suggests the development of hydrogen bonds between the carriers, thus ensuring the encapsulation of the essential oil and, consequently, its protection against evaporation and oxidation. Τhe possibility of hydrogen bond formation is further strengthened by the slight shift of the above-mentioned peak noticed in the spectra of OEO-loaded particles (i.e., WM: from 3358 to 3355 cm^−1^, W3M: from 3366 to 3370 cm^−1^).

### 3.7. Antibacterial Activity

Disc diffusion zones, expressed as zone diameters, were obtained for the bacterial strains *E. coli* and *S. aureus* using pure OEO, OEO-containing, and empty spray dried powders. The results are summarized in Table 4. Pure OEO was tested in four different concentrations to simulate the corresponding EO concentrations of the encapsulated samples (25 and 75 mg). Its antibacterial activity towards the tested microorganisms was confirmed, while no significant differences were noted in relation to EO concentration increase. Spray-dried powders without EO (only wall material) were also exposed to the same strains and no presence of inhibition zones was noticed for all the tested amounts (25–125 mg) and for both samples (WM and W3M). Hence, the presence of OEO was considered responsible for the formed zones.

Both samples were active against the studied microorganisms, accomplishing high levels of antibacterial activity even at relatively small amounts of core material, with that of W3M appearing more efficient (Table 4). Specifically, the inhibition zones of *E. coli* were in the range of 30.0–42.3 mm and 33.0–41.3 mm for the WM and W3M, respectively. Higher susceptibility was demonstrated by *S. aureus*, achieving zone diameters of 36.3–51.0 and 37.0–48.7 mm for WM and W3M, respectively. The greater sensitiveness of gram-positive bacteria to spray dried powders in comparison to that of gram negative was, also, stated by Keawchaoon and Yoksan (2011) [41] and could be attributed to the composition of gram-negative bacteria’s outer membrane that contains hydrophilic lipopolysaccharides able to restrict the diffusion of hydrophobic compounds [49]. In consequence, a greater concentration of the antimicrobial agent is necessary to acquire the same effect as that of gram-positive bacteria.

It is worth noticing that by increasing the powder amount, and accordingly the OEO concentration, a decrease in inhibition zone diameters was detected for all the samples, though without showing any statistically significant differences in some cases. This phenomenon could be attributed to the fact that a small volume is entrapped in a too high amount of wall material, thus resulting in a supersaturation of EO that blocks its release. A similar situation has been referred to by Anaya-Castro et al. (2017) [50] while testing the antimicrobial activity of OEO encapsulated in *β*-cyclodextrin inclusion complexes. After one month of refrigerated storage, the antibacterial effect of OEO-loaded particles was positively affected (Table 4). This could possibly be due to the gradual release of core material to the particles’ external areas during storage.

EO’s antibacterial activity was not only preserved, but also enhanced with the encapsulation in WPI-MD matrices by applying the Nano SD technique. This increment could be related to the controlled release that was achieved as a result of the encapsulation procedure [15] or to the large surface of the encapsulated material arising from the very small size, favoring interactions with the bacteria’s cell surfaces [51]. Foregoing works applying different methods for OEO encapsulation, such as encapsulation in *β*-cyclodextrin inclusion complexes by co-precipitation method [50], spray drying [7], and nanoemulsification [16], demonstrated that encapsulation did not affect EO’s antimicrobial activity, but improved it. Overall, the obtained results confirmed that OEO processing via emulsification and NSD can preserve its antibacterial characteristics.

## 4. Conclusions

Encapsulation of OEO in whey protein isolate-maltodextrin system was successfully achieved by nano spray drying, a novel technique that achieves much higher yields than the conventional one. OEO particles were of fine size and spherical shape, presented remarkable encapsulation efficiency, satisfactory physical properties, and great potential to preserve essential oil’s main components, indicating good core protection. Moreover, their increased antibacterial activity in comparison with that of pure OEO makes their use as preservation agents in food, cosmetics, and in the medical industry very likely. The results of this work uncovered the potential of nano spray drying as a good alternative to standard spray drying in formulating dry powders of OEO. Nevertheless, the low throughput and the increased processing hours are drawbacks that still concern researchers.

## Figures and Tables

**Table 1:** Composition (*w*/*w*) of OEO emulsions and powder recovery after spray drying.

Samples	OEO	WPI	MD	Powder Recovery ^1^ (%)
WM	5	10	10	81.58 ± 1.78 ^b^
W3M	5	5	15	77.93 ± 1.51 ^a^

^1^ Mean ± SD (*n* = 5); values within columns followed by different superscripts represent significant differences (*p* < 0.05).

**Table 2:** Properties of oregano essential oil particles.

	WPI:MD	
	1:1	1:3
Total oil content (%)	10.98 ± 0.29 ^b^	9.88 ± 0.36 ^a^
Surface oil content (%)	0.21 ± 0.03 ^a^	0.93 ± 0.16 ^b^
Oil retention (%)	54.88 ± 1.44 ^b^	49.39 ± 1.80 ^a^
Encapsulation efficiency (%)	98.06 ± 0.29 ^b^	90.58 ± 1.48 ^a^
Moisture (%)	9.23 ± 0.65 ^a^	8.19 ± 0.37 ^a^
Bulk density (g/cm^3^)	0.17 ± 0.01 ^a^	0.20 ± 0.01 ^b^
Hygroscopicity (%)	14.76 ± 0.09 ^a^	15.78 ± 0.99 ^a^
Dispersibility (%)	57.10 ± 1.62 ^a^	64.17 ± 1.84 ^b^
Wettability (min)	7.28 ± 0.20 ^b^	5.19 ± 0.07 ^a^

Data are represented as means ± SD (*n* = 3); values within rows followed by different superscripts represent significant differences (*p* < 0.05).

**Table 3:** Levels of main components of OEO before and after encapsulation, expressed as peak area (%) for the major components identified by GC-FID.

		OEO Encapsulated in	
	Pure OEO	WPI:MD	WPI:3MD
		Total	Total
Myrcene	1.50 ± 0.06	nd	nd
α-terpinene	1.66 ± 0.05	nd	nd
γ-terpinene	5.20 ± 0.19 ^b^	2.74 ± 0.07 ^a^	2.94 ± 0.79 ^a^
p-cymene	6.06 ± 0.21 ^b^	2.33 ± 0.33 ^a^	2.41 ± 0.30 ^a^
β-caryophyllene	2.17 ± 0.03 ^a^	4.31 ± 0.05 ^b^	4.44 ± 0.56 ^b^
Terpinen-4-ol	0.52 ± 0.07 ^b^	0.21 ± 0.03 ^a^	0.29 ± 0.12 ^a^
Thymol	1.38 ± 0.00 ^b^	1.29 ± 0.00 ^a^	1.28 ± 0.04 ^a^
Carvacrol	80.03 ± 0.35 ^a^	86.64 ± 0.30 ^b^	86.05 ± 1.82 ^b^
Others	1.48 ± 0.14 ^a^	2.48 ± 0.05 ^b^	2.59 ± 0.35 ^b^

Data are presented as means ± SD (*n* = 5); values within rows followed by different superscripts represent significant differences (*p* < 0.05).

**Table 4:** Antibacterial activities (measured as inhibition zone diameters in mm) of OEO-loaded particles and pure OEO against *E. coli* and *S. aureus*.

		Inhibition Zone Diameter (mm) ^1^									
		**WM**					**W3M**				
	Sample weight (mg)	25	50	75	100	125	25	50	75	100	125
	OEO in test discs (mg)	2.75	5.49	8.24	10.98	13.73	2.47	4.94	7.11	9.88	12.35
*E. coli*	t = 0	42.3 ± 3.8 ^b^	31.7 ± 1.5 ^a^	31.0 ± 1.0 ^a^	30.3 ± 1.5 ^a^	30.0 ± 2.0 ^a^	41.3 ± 1.5 ^c^	37.0 ± 2.0 ^b^	36.7 ± 1.5 ^a,b^	33.0 ± 2.0 ^a^	34.0 ± 2.7 ^a,b^
	t = 1 month	50.7 ± 2.1 ^b^	-	46.0 ± 1.7 ^a^	-	-	51.7 ± 2.9 ^a^	-	47.3 ± 2.3 ^a^	-	-
*S. aureus*	t = 0	51.0 ± 1.0 ^c^	43.7 ± 1.5 ^b^	38.3 ± 3.1 ^a^	36.7 ± 1.2 ^a^	36.3 ± 0.6 ^a^	48.7 ± 3.1 ^d^	44.0 ± 2.0 ^b,c^	44.7 ± 1.5 ^c^	40.3 ± 1.5 ^a,b^	37.0 ± 1.7 ^a^
	t = 1 month	51.7 ± 2.9 ^b^	-	47.0 ± 2.0 ^a^	-	-	54.0 ± 3.6 ^b^	-	48.0 ± 2.6 ^a^	-	-
		**Pure OEO**					**Pure OEO**				
	OEO in test discs (mg)	2.75			8.24		2.47			7.11	
*E. coli*		34.3 ± 3.8 ^a^			35.0 ± 1.0 ^a^		39.7 ± 2.5 ^a^			38.7 ± 1.5 ^a^	
*S. aureus*		44.7 ± 1.5 ^a^			45.7 ± 2.1 ^a^		46.0 ± 1.7 ^a^			45.7 ± 1.2 ^a^	

^1^ Values represent the average of three replicates ± SD. ^a–d^ Superscripts with the same letters in the same line, for each sample separately, were not significantly different (*p* > 0.05).

## Data Availability

Not applicable.

## Supplementary Materials

The following are available online at https://www.mdpi.com/article/10.3390/foods10122923/s1, Figure S1: Droplet size distribution of OEO emulsions after four days storage.

## Abbreviations

OEO	oregano essential oil
NSD	nano spray drying
WPI	whey protein isolate
MD	maltodextrin
EO	essential oil
DE	dextrose equivalent
PDI	polydispersity index
TO	total oil content
SO	surface oil content
*OR*	oil retention
*EE*	encapsulation efficiency
SMP	skim milk powder
MSD	conventional/mini spray drying
MS	modified starch
GA	gum Arabic

## References

[1] Majeed H., Bian Y.-Y., Ali B., Jamil A., Majeed U., Khan Q.F., Iqbal K.J., Shoemaker C.F., Fang Z. (2015). Essential oil encapsulations: Uses, procedures, and trends. RSC Adv..

[2] Asensio C.M., Paredes A.J., Martin M.P., Allemandi D.A., Nepote V., Grosso N.R. (2017). Antioxidant stability study of oregano essential oil microcapsules prepared by spray-drying. J. Food Sci..

[3] Arana-Sánchez A., Estarrón-Espinosa M., Obledo-Vázquez E.N., Padilla-Camberos E., Silva-Vázquez R., Lugo-Cervantes E. (2010). Antimicrobial and antioxidant activities of Mexican oregano essential oils (*Lippia graveolens* H. B. K.) with different composition when microencapsulated in β-cyclodextrin. Lett. Appl. Microbiol..

[4] Bakry A.M., Abbas S., Ali B., Majeed H., Abouelwafa M.Y., Mousa A., Liang L. (2016). Microencapsulation of oils: A comprehensive review of benefits, techniques, and applications. Compr. Rev. Food Sci. Food Saf..

[5] Sivropoulou A., Papanikolaou E., Nikolaou C., Kokkini S., Lanaras T., Arsenakis M. (1996). Antimicrobial and cytotoxic activities of Origanum essential oils. J. Agric. Food Chem..

[6] Jafari S.M., Assadpoor E., He Y., Bhandari B. (2008). Encapsulation efficiency of food flavours and oils during spray drying. Dry. Technol..

[7] Partheniadis I., Vergkizi S., Lazari D., Reppas C., Nikolakakis I. (2019). Formulation, characterization and antimicrobial activity of tablets of essential oil prepared by compression of spray-dried powder. J. Drug Deliv. Sci. Technol..

[8] Botrel D.A., Vilela Borges S., Victória de Barros Fernandes R., Dantas Viana A., Maria Gomes da Costa J., Reginaldo Marques G. (2012). Evaluation of spray drying conditions on properties of microencapsulated oregano essential oil. Int. J. Food Sci. Technol..

[9] Baranauskaite J., Ivanauskas L., Masteikova R., Kopustinskiene D., Baranauskas A., Bernatoniene J. (2016). Formulation and characterization of Turkish oregano microcapsules prepared by spray-drying technology. Pharm. Dev. Technol..

[10] Da Costa J.M.G., Borges S.V., Hijo A.A.C.T., Silva E.K., Marques G.R., Cirillo M.Â., De Azevedo V.M. (2013). Matrix structure selection in the microparticles of essential oil oregano produced by spray dryer. J. Microencapsul..

[11] Toledo Hijo A.A.C., Da Costa J.M.G., Silva E.K., Azevedo V.M., Yoshida M.I., Borges S.V. (2015). Physical and thermal properties of oregano (*Origanum vulgare* L.) essential oil microparticles. J. Food Process. Engin..

[12] Baranauskaite J., Kopustinskiene D.M., Bernatoniene J. (2019). Impact of gelatin supplemented with gum Arabic, Tween 20, and β-cyclodextrin on the microencapsulation of Turkish oregano extract. Molecules.

[13] Baranauskiene R., Venskutonis P.R., Dewettinck K., Verhé R. (2006). Properties of oregano (*Origanum vulgare* L.), citronella (*Cymbopogon nardus* G.) and marjoram (*Majorana hortensis* L.) flavors encapsulated into milk protein-based matrices. Food Res. Int..

[14] Rodríguez J., Martín M.J., Ruiz M.A., Clares B. (2016). Current encapsulation strategies for bioactive oils: From alimentary to pharmaceutical perspectives. Food Res. Int..

[15] Hosseini S.F., Zandi M., Rezaei M., Farahmandghavi F. (2013). Two-step method for encapsulation of oregano essential oil in chitosan nanoparticles: Preparation, characterization and in vitro release study. Carbohyd. Polym..

[16] Dávila-Rodríguez M., López-Malo A., Palou E., Ramírez-Corona N., Jiménez-Munguía M.T. (2019). Antimicrobial activity of nanoemulsions of cinnamon, rosemary, and oregano essential oils on fresh celery. Lwt—Food Sci. Technol..

[17] Gonçalves da Rosa C.G., Zapelini de Melo A.P., Sganzerla W.G., Machado M.H., Nunes M.R., Maciel M.V.D.O.B., Bertoldi F.C., Manique Barreto P.L. (2020). Application in situ of zein nanocapsules loaded with *Origanum vulgare* Linneus and *Thymus vulgaris* as a preservative in bread. Food Hydrocoll..

[18] Yilmaz M.T., Yilmaz A., Akman P.K., Bozkurt F., Dertli E., Basahel A., Al-Sasi B., Taylan O., Sagdic O. (2019). Electrospraying method for fabrication of essential oil loaded-chitosan nanoparticle delivery systems characterized by molecular, thermal, morphological and antifungal properties. Innov. Food Sci. Emerg. Technol..

[19] Kotronia M., Kavetsou E., Loupassaki S., Kikionis S., Vouyiouka S., Detsi A. (2017). Encapsulation of oregano (*Origanum onites* L.) essential oil in β-cyclodextrin (β-CD): Synthesis and characterization of the inclusion complexes. Bioengineering.

[20] Hernández-Nava R., López-Malo A., Palou E., Ramírez-Corona N., Jiménez-Munguía M.T. (2020). Encapsulation of oregano essential oil (*Origanum vulgare*) by complex coacervation between gelatin and chia mucilage and its properties after spray drying. Food Hydrocoll..

[21] De Medeiros J.A.S., Blick A.P., Galindo M.V., Alvim I.D., Yamashita F., Ueno C.T., Shirai M.A., Grosso C.R.F., Corradini E., Sakanaka L.S. (2019). Incorporation of oregano essential Oil microcapsules in starch-poly (Butylene Adipate Co-Terephthalate) (PBAT) films. Macromol. Symp..

[22] Wu Z., Zhou W., Pang C., Deng W., Xu C., Wang X. (2019). Multifunctional chitosan-based coating with liposomes containing laurel essential oils and nanosilver for pork preservation. Food Chem..

[23] Lee S.H., Heng D., Ng W.K., Chan H.K., Tan R.B.H. (2011). Nano spray drying: A novel method for preparing protein nanoparticles for protein therapy. Int. J. Pharm..

[24] Arpagaus C. (2012). A novel laboratory-scale spray dryer to produce nanoparticles. Dry. Technol..

[25] Piñón-Balderrama C.I., Leyva-Porras C., Terán-Figueroa Y., Espinosa-Solis V., Alvarez-Salas C., Saavedra-Leos M. (2020). Encapsulation of active ingredients in food industry by spray-drying and encapsulation of active ingredients in food industry by spray-drying and nano spray-drying technologies. Processes.

[26] Stavra K., Plati F., Pavlidou E., Paraskevopoulou A. (2021). Characterization of lemon juice powders produced by different drying techniques and carrier materials. Dry. Technol..

[27] Heng D., Lee S.H., Ng W.K., Tan R.B. (2011). The nano spray dryer B-90. Expert Opin. Drug Deliv..

[28] Bae E.K., Lee S.J. (2008). Microencapsulation of avocado oil by spray drying using whey protein and maltodextrin. J. Microcapsul..

[29] de Barros Fernandes R.V., Silva E.K., Borges S.V., de Oliveira C.R., Yoshida M.I., da Silva Y.F., do Carmo E.L., Azevedo V.M., Botrel D.A. (2017). Proposing novel encapsulating matrices for spray-dried ginger essential oil from the whey protein isolate-inulin/maltodextrin blends. Food Bioprocess. Technol..

[30] Botrel D.A., de Barros Fernandes R.V., Borges S.V., Yoshida M.I. (2014). Influence of wall matrix systems on the properties of spray-dried microparticles containing fish oil. Food Res. Int..

[31] Campelo P.H., do Carmo E.L., Zacarias R.D., Yoshida M.I., Ferraz V.P., de Barros Fernandes R.V., Botrel D.A., Borges S.V. (2017). Effect of dextrose equivalent on physical and chemical properties of lime essential oil microparticles. Ind. Crops Prod..

[32] Gharsallaoui A., Roudaut G., Chambin O., Voilley A., Saurel R. (2007). Applications of spray-drying in microencapsulation of food ingredients: An overview. Food Res. Int..

[33] Hu Q., Gerhard H., Upadhyaya I., Venkitanarayanan K., Luo Y. (2016). Antimicrobial eugenol nanoemulsion prepared by gum arabic and lecithin and evaluation of drying technologies. Int. J. Biolog. Macromol..

[34] Veneranda M., Hu Q., Wang T., Luo Y., Castro K., Madariaga J.M. (2018). Formation and characterization of zein-caseinate-pectin complex nanoparticles for encapsulation of eugenol. LWT—Food Sci. Technol..

[35] Kokkini S., Karousou R., Hanlidou E., Lanaras T. (2004). Essential oil composition of greek (*Origanum vulgare* ssp. *hirtum*) and turkish (*O. onites*) oregano: A tool for their distinction. J. Essent. Oil Res..

[36] AOAC (2012). Official Methods of Analysis, Association of Official Analytical Chemist.

[37] Plati F., Matsakidou A., Kiosseoglou V., Paraskevopoulou A. (2019). Development of a dehydrated dressing-type emulsion with instant powder characteristics. Food Struct..

[38] Cai Y.Z., Corke H. (2000). Production and properties of spray-dried Amaranthus betacyanin pigments. J. Food Sci..

[39] Bauer A.W., Kirby W.M., Sherris J.C., Turck M. (1966). Antibiotic susceptibility testing by a standardized single disk method. Am. J. Clin. Pathol..

[40] Andriotis E.G., Papi R.M., Paraskevopoulou A., Achilias D.S. (2021). Synthesis of d-limonene loaded polymeric nanoparticles with enhanced antimicrobial properties for potential application in food packaging. Nanomaterials.

[41] Keawchaoon L., Yoksan R. (2011). Preparation, characterization and in vitro release study of carvacrol-loaded chitosan nanoparticles. Colloids Surf. B.

[42] Shamaei S., Seiiedlou S.S., Aghbashlo M., Tsotsas E., Kharaghani A. (2017). Microencapsulation of walnut oil by spray drying: Effects of wall material and drying conditions on physicochemical properties of microcapsules. Innov. Food Sci. Emerg. Technol..

[43] Goula A.M., Adamopoulos K.G. (2010). A new technique for spray drying orange juice concentrate. Innov. Food Sci. Emerg. Technol..

[44] Kausadikar S., Gadhave A.D., Waghmare J. (2015). Microencapsulation of lemon oil by spray drying and its application in flavour tea. Adv. Appl. Sci. Res..

[45] Victória R., Fernandes D.B., Borges S.V., Botrel A., Silva E.K., Maria J., Botrel A., Silva E.K., Maria J., Queiroz F. (2013). Microencapsulation of rosemary essential oil: Characterization of particles. Dry. Technol..

[46] Koupantsis T., Pavlidou E., Paraskevopoulou A. (2016). Glycerol and tannic acid as applied in the preparation of milk proteins—CMC complex coavervates for flavour encapsulation. Food Hydrocoll..

[47] Tavares L., Noreña C.P.Z. (2020). Encapsulation of ginger essential oil using complex coacervation method: Coacervate formation, rheological property, and physicochemical characterization. Food Bioprocess. Technol..

[48] Kang Y.R., Lee Y.K., Kim Y.J., Chang Y.H. (2019). Characterization and storage stability of chlorophylls microencapsulated in different combination of gum Arabic and maltodextrin. Food Chem..

[49] Burt S. (2004). Essential oils: Their antibacterial properties and potential applications in foods—A review. Int. J. Food Microbiol..

[50] Anaya-Castro M.A., Ayala-Zavala J.F., Muñoz-Castellanos L., Hernández-Ochoa L., Peydecastaing J., Durrieu V. (2017). β-Cyclodextrin inclusion complexes containing clove (*Eugenia caryophyllata*) and Mexican oregano (*Lippia berlandieri*) essential oils: Preparation, physicochemical and antimicrobial characterization. Food Packag. Shelf Life.

[51] Lboutounne H. (2002). Sustained ex vivo skin antiseptic activity of chlorhexidine in poly (e-caprolactone) nanocapsule encapsulated form and as a digluconate. J. Control. Release.

