# Supplementary material for: Characterization of Oregano Essential Oil (Origanum vulgare L. subsp. hirtum) Particles Produced by the Novel Nano Spray Drying Technique
Source: Foods. 2021 Nov 25;10(12):2923. doi: 10.3390/foods10122923 (PMC8700915; doi:10.3390/foods10122923)
Supplement: Supplementary file 1 [file foods-10-02923-s001.zip › foods-1449070-supplementary.pdf]

Characterization of Oregano Essential Oil (*Origanum vulgare* L. subsp. *hirtum*) Particles Produced by the Novel Nano Spray Drying Technique

Fotini Plati [1], Rigini Papi [2] and Adamantini Paraskevopoulou [1,*]

[1] Laboratory of Food Chemistry and Technology, School of Chemistry, Aristotle University of Thessaloniki,
54 124 Thessaloniki, Greece
[2] Laboratory of Biochemistry, School of Chemistry, Aristotle University of Thessaloniki, 54 124 Thessaloniki, Greece
* Correspondence: adparask@chem.auth.gr

Supplemmentary Material

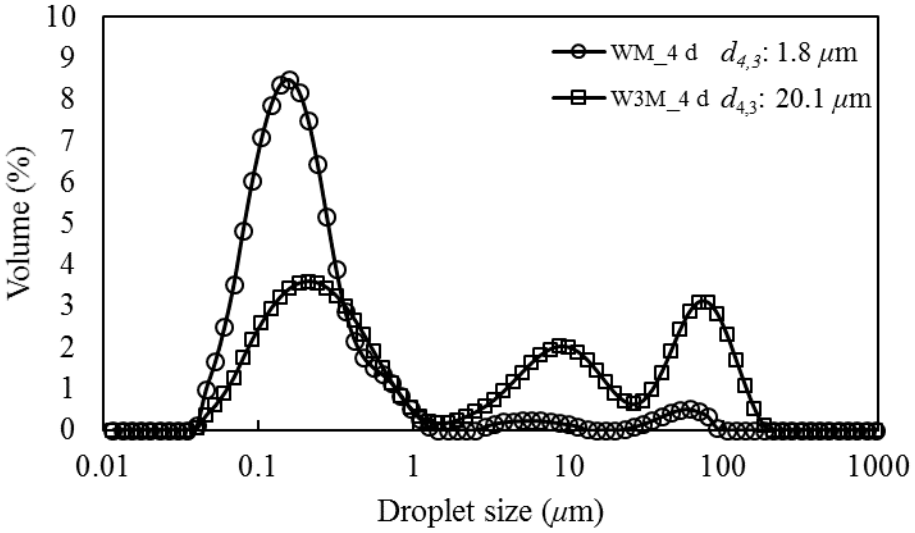

Figure S1. Droplet size distribution of OEO emulsions after four days storage.
